# The association between smoking cessation before and after diagnosis and non-muscle-invasive bladder cancer recurrence: a prospective cohort study

**DOI:** 10.1007/s10552-018-1046-8

**Published:** 2018-05-30

**Authors:** Frits H. M. van Osch, Sylvia H. J. Jochems, Raoul C. Reulen, Sarah J. Pirrie, Duncan Nekeman, Anke Wesselius, Nicholas D. James, D. Michael A. Wallace, K. K. Cheng, Frederik J. van Schooten, Richard T. Bryan, Maurice P. Zeegers

**Affiliations:** 1 Unit of Nutritional and Cancer Epidemiology, Chairgroup of Complex Genetics and Epidemiology, School for Nutrition and Translational Research in Metabolism (NUTRIM), Maastricht University, Maastricht, The Netherlands; 2 Institute of Cancer and Genomic Sciences, University of Birmingham, Birmingham, UK; 3 Department of Public Health and Epidemiology, University of Birmingham, Birmingham, UK; 4 University Hospital Birmingham, NHS Foundation Trust, Birmingham, UK; 5 Department of Pharmacology and Toxicology, School for Nutrition and Translational Research in Metabolism (NUTRIM), Maastricht University, Maastricht, The Netherlands; 6 Chairgroup of Complex Genetics and Epidemiology, Care and Public Health Research Institute (CAPRHI), Maastricht University, Maastricht, The Netherlands

**Keywords:** Smoking, Smoking cessation, Bladder cancer, Prognosis, Recurrence, Epidemiology

## Abstract

**Background:**

Smoking is a major risk factor for bladder cancer, but the relationship between smoking cessation after initial treatment and bladder cancer recurrence has been investigated less frequently and not prospectively yet.

**Methods:**

722 non-muscle-invasive bladder cancer (NMIBC) patients (pTa, pT1, and CIS) from the prospective Bladder Cancer Prognosis Programme (BCPP) cohort, selected in the UK between 2005 and 2011, provided complete data on smoking behavior before and up to 5 years after diagnosis. The impact of smoking behavior on NMIBC recurrence was explored by multivariable Cox regression models investigating time-to-first NMIBC recurrence.

**Results:**

Over a median follow-up period of 4.21 years, 403 pathologically confirmed NMIBC recurrences occurred in 210 patients. Only 25 current smokers at diagnosis quit smoking (14%) during follow-up and smoking cessation after diagnosis did not decrease risk of recurrence compared to continuing smokers (*p* = 0.352).

**Conclusions:**

Although quitting smoking after diagnosis might reduce the risk of recurrence based on retrospective evidence, this is not confirmed in this prospective study because the number of NMIBC patients quitting smoking before their first recurrence was too low. Nevertheless, this indicates an important role for urologists and other health care professionals in promoting smoking cessation in NMIBC.

## Introduction

Bladder cancer (BC) is estimated to be the ninth most frequent cancer worldwide with approximately 400,000 newly diagnosed cases per year [1]. Compared to other cancers, mortality rates are generally lower for BC [1] since the majority of BCs diagnosed are non-muscle-invasive bladder cancers (NMIBC) [2]. However, NMIBC often recurs [3] and has a risk of progressing to muscle-invasive bladder cancer (MIBC) [4], events which impact on the quality of life of the patient [5] and generate high disease management costs [6].

Although smoking is an established risk factor for BC, its effects has been less frequently investigated in relation to BC prognosis [7–10]. Although many studies investigated effectiveness of treatment for NMIBC and MIBC with regard to recurrence, progression, and mortality, most studies did not investigate the effect of smoking or other factors modifiable by patients on BC prognosis [11]. Nevertheless, the number of studies also reporting hazard ratios (HRs) for BC recurrence by smoking status at diagnosis has increased recently and the current body of evidence consistently shows that there is a small association between smoking and BC recurrence when comparing current smokers to never smokers at diagnosis [10, 12]. However, the impact of smoking cessation after BC diagnosis on recurrence and mortality has not yet been quantified prospectively [13]. Studies have investigated the impact of smoking cessation within 1 year after diagnosis on BC recurrence, showing a slight decrease in risk of recurrence [14, 15], and one study indicates no effect of quitting after diagnosis on overall or bladder cancer-specific mortality [16].

The Bladder Cancer Prognosis Programme (BCPP) followed up BC patients for 5 years post-diagnosis and investigated changes in smoking behavior in relation to the course of the disease [17]. The principal aim of this study was to investigate whether smoking cessation post-diagnosis and smoking behavior pre-diagnosis influences BC recurrence.

## Methods

### The Bladder Cancer Prognosis programme

This study was conducted within the framework of the West Midlands Bladder Cancer Prognosis Programme (BCPP), a cohort study in the United Kingdom. Details of the study are described elsewhere [17]. In brief, individuals were included between December 2005 and October 2011 after referral to participating urology centers due to symptoms suspicious of BC and followed for a maximum of 5 years from diagnosis. Patients with previous cancer of the urethra, bladder, ureter, or renal pelvis within the last decade were excluded. The study was ethically approved (06/MRE04/65) and all participants gave written informed consent.

### Data collection

At or around time of diagnosis, trained research nurses used semi-structured face-to-face interviews and questionnaires to collect data on social support, health-related quality of life, sociodemographics, medical history, and health-related behaviors including smoking behavior. Variables on smoking behavior included current smoking status (never, former, current), duration (years of smoking), intensity (cigarettes per day), smoking cessation (in years), and tobacco type (filter, non-filter, or rolled cigarettes, cigar, or pipe). Monthly smoking status was also assessed retrospectively by postal questionnaires that were sent out to participants yearly until the end of follow-up.

### Smoking status at diagnosis and during follow-up

A combined smoking status variable was created indicating continuing smokers, former smokers who consistently abstained, never smokers, former smokers who started smoking again, and current smokers who quit smoking post-diagnosis. Patients were considered quitters when they abstained consistently, so smokers who quit for 3 months and then started again were considered as continuing smokers. Furthermore, for each participant that reported smoking cessation during follow-up, it was confirmed whether this occurred before or after their first recurrence. If patients quit smoking after their first recurrence, they were considered as continuing smokers in the time-to-first recurrence analysis.

### Population at risk

Of the 1,550 cases who agreed to participate, 231 were subsequently identified as not having BC. Patients who presented with MIBC (*n* = 275) disease at diagnosis were excluded from analysis because they are fundamentally different from NMIBC with regard to recurrence. Patients with squamous or adenocarcinomas of non-urothelial origin or with bladder cancer as secondary carcinoma were excluded (*n* = 41). In addition to patients presenting with Ta and T1 tumors, carcinoma in situ (CIS) tumors were included (*n* = 16) since they have an increased risk of recurrence [18]. In total, 846 (84%) of these patients had provided data on smoking behavior at diagnosis and during follow-up and remained under follow-up within the cohort study. Of the included 846 NMIBC patients, there were 116 patients with unknown recurrent tumor stage. These 116 unconfirmed events were excluded for other analyses as well as 8 cases who had radiotherapy (on suspicion of being MIBC cases) resulting in a NMIBC patient population at risk of recurrence of 722.

No systematic guidance or tools were provided to enable patients to quit smoking after diagnosis, so care as usual was applied by all participating urologists.

### Statistical analysis

BC recurrence was defined as a new tumor that was at the same stage as the primary tumor (Ta or T1) but also when a primary Ta patient had a T1 recurrence. Patients that progressed from T1 to T2 disease were not counted as a recurrence but as a progression event. Unfortunately, there were not enough events to also consider biological progression within this sample of NMIBC patients, as defined in the BCPP cohort [19]. Therefore, this study only focussed on confirmed recurrence events and patients who experienced a progression event were censored in the survival analysis when the progression event was diagnosed.

The impact of smoking behavior on BC recurrence was explored by Cox regression models—with time since initial transurethral resection of the bladder tumor (TURBT) as the time-metric—investigating possible differences in likelihood of a first recurrence. We explored two different Cox regression models: one adjusted for age at diagnosis and sex (model 1) and one additionally adjusted for BC stage, grade, tumor size, and number of tumors at diagnosis (model 2). This set of confounders was chosen since they are markers of NMIBC prognosis and are factors that contribute to European Association of Urology (EAU) risk stratification for clinical decisions [20]. Moreover, they are potentially associated with smoking behavior at diagnosis [21]. Consequently, conditional risk set modeling was applied to investigate time between multiple recurrent events and analysis time was reset at each event [22]. For this analysis, re-resection of tumors was added to model 2 as a confounder. The proportional hazards assumption was checked in all models using Schoenfeld residuals. Cumulative incidence functions (CIF) corrected for competing risks (death) were made [23].

Furthermore, the differences in mean number of recurrences over 5 years between never smokers, former smokers, and continuing smokers were compared using a multivariable ANOVA model correcting for pairwise comparisons using Tukey’s HSD. There were not enough BC-related death events (45) or confirmed progression events (19) to allow for separate analyses. A similarly low number of progression events has been observed in a large (*n* = 718) NMIBC patient sample before [24].

NMIBC patients who died before the end of follow-up (*n* = 157) were censored at time of death, and patients who underwent cystectomy (*n* = 15) were censored at the date of cystectomy (13). Other patients were considered lost to follow-up when the date on which patients were last seen in the hospital for bladder cancer-related therapy or the date on which they filled in their last follow-up questionnaire was before the end of follow-up (5 years).

## Results

### Number of recurrences and characteristics of population at risk

All 722 patients at risk of recurrence were followed over a median period of 4.21 years (IQR 2.64–5.00 years). The majority of patients (506, 70%) were followed for at least 3 years. Over this period of follow-up, 210 NMIBC patients experienced at least one confirmed recurrence event. These 210 NMIBC patients accumulated a total of 403 confirmed recurrence events in the cohort.

Most cases were male (79%) and around the age of 70 (Table 1). Furthermore, continuing smokers seemed to be underrepresented in the low EAU risk group (12%), those who quit smoking seemed more likely to be younger and female, and continuing smokers seemed more likely to present with multiple tumors at diagnosis (Table 1). In the multivariate models, 26 patients were not included in the analysis due to missing data on age (*n* = 7), number of tumors at diagnosis (*n* = 15), and tumor size (*n* = 4). Because participants were recruited from multiple centers, patients were treated by multiple urologists with different individual thresholds to perform certain therapies. Therefore, not all patients were treated exactly according to the EAU guidelines [20], which is often the case in actual clinical practice [25].

**Table 1:** Patient characteristics at diagnosis and number of recurrences over 5 years for 722 NMIBC patients treated with transurethral resection by smoking category

	Overall (*n* = 722)	Combined smoking status					
		Never smoker (*n* = 103)	Former smoker (*n* = 266)	Continuing smoker (*n* = 186)	Former smoker who started again (*n* = 150)	Quitters after diagnosis (*n* = 17)	*p* value*
Age in years							< 0.001
Median (25th–75th percentile)	71 (63–77)	72 (61–79)	72 (67–79)	67 (57–74)	72 (64–77)	62 (56–67)	
Sex							< 0.001
Male	573 (79%)	63 (61%)	231 (87%)	139 (75%)	129 (86%)	11 (65%)	
Female	149 (21%)	40 (39%)	35 (13%)	47 (25%)	21 (14%)	6 (35%)	
EAU risk group							< 0.001
Low	128 (18%)	28 (27%)	71 (27%)	23 (12%)	4 (3%)	2 (12%)	
Intermediate	383(53%)	50 (49%)	131 (49%)	97 (52%)	91 (61%)	14 (82%)	
High	211 (29%)	25 (24%)	64 (24%)	66 (36%)	55 (37%)	1 (6%)	
Number of tumors							< 0.001
1	429 (61%)	70 (70%)	179 (69%)	100 (55%)	69 (46%)	11 (65%)	
2–7	258 (36%)	27 (27%)	74 (28%)	76 (42%)	75 (50%)	6 (35%)	
≥ 8	22 (3%)	3 (3%)	8 (3%)	6 (3%)	5 (3%)	0 (-)	
Tumor size							0.068
< 3 cm	445 (63%)	68 (68%)	174 (67%)	105 (58%)	85 (57%)	13 (76%)	
≥ 3 cm	260 (37%)	32 (32%)	84 (33%)	77 (42%)	63 (43%)	4 (24%)	
Grade							0.001
1	212 (30%)	34 (34%)	99 (38%)	51 (28%)	26 (17%)	2 (13%)	
2	257 (36%)	34 (34%)	75 (28%)	73 (40%)	66 (44%)	9 (56%)	
3	245 (34%)	33 (33%)	90 (34%)	60 (32%)	57 (38%)	5 (31%)	
Stage							0.590
pTa	476 (66%)	68 (66%)	184 (69%)	115 (62%)	95 (63%)	14 (82%)	
pT1	239 (33%)	35 (34%)	79 (30%)	69 (37%)	53 (35%)	3 (18%)	
pCis	7 (1%)	0 (-)	3 (1%)	2 (1%)	2 (1%)	0 (-)	
No of recurrences							0.337
1	108 (51%)	18 (62%)	28 (46%)	33 (53%)	27 (52%)	2 (33%)	
2	46 (22%)	6 (21%)	16 (26%)	16 (26%)	6 (11%)	2 (33%)	
> 3	56 (27%)	5 (17%)	17 (28%)	13 (21%)	19 (37%)	2 (33%)	
Smoking intensity							0.076
1–9 cigarettes	128 (29%)	NA	55 (30%)	23 (21%)	42 (34%)	8 (50%)	
10–19 cigarettes	140 (32%)	NA	53 (28%)	42 (38%)	42 (34%)	3 (19%)	
>20 cigarettes	167 (38%)	NA	78 (42%)	45 (41%)	39 (32%)	5 (31%)	
Smoking duration							< 0.001
1–9 years	45 (10%)	NA	26 (14%)	2 (2%)	16(14%)	1 (6%)	
10–19 years	83 (19%)	NA	43 (23%)	10 (9%)	29 (25%)	1 (6%)	
20–29 years	87 (20%)	NA	46 (25%)	12 (11%)	27 (23%)	2 (13%)	
30–39 years	88 (21%)	NA	37 (20%)	28 (25%)	19 (16%)	4 (25%)	
> 40 years	127 (30%)	NA	32 (17%)	60 (54%)	27 (23%)	8 (50%)	
Smoking cessation							0.051
< 20 years	48 (12%)	NA	23 (9%)	NA	25 (17%)	NA	
21–40 years	208 (51%)	NA	134 (51%)	NA	74 (49%)	NA	
> 40 years	155 (38%)	NA	104 (40%)	NA	51 (34%)	NA	

*Kruskal–Wallis test for continuous and Chi-square test for categorical variables

### Associations between smoking behavior pre- and post-diagnosis and BC recurrence

**Fig. 1:**
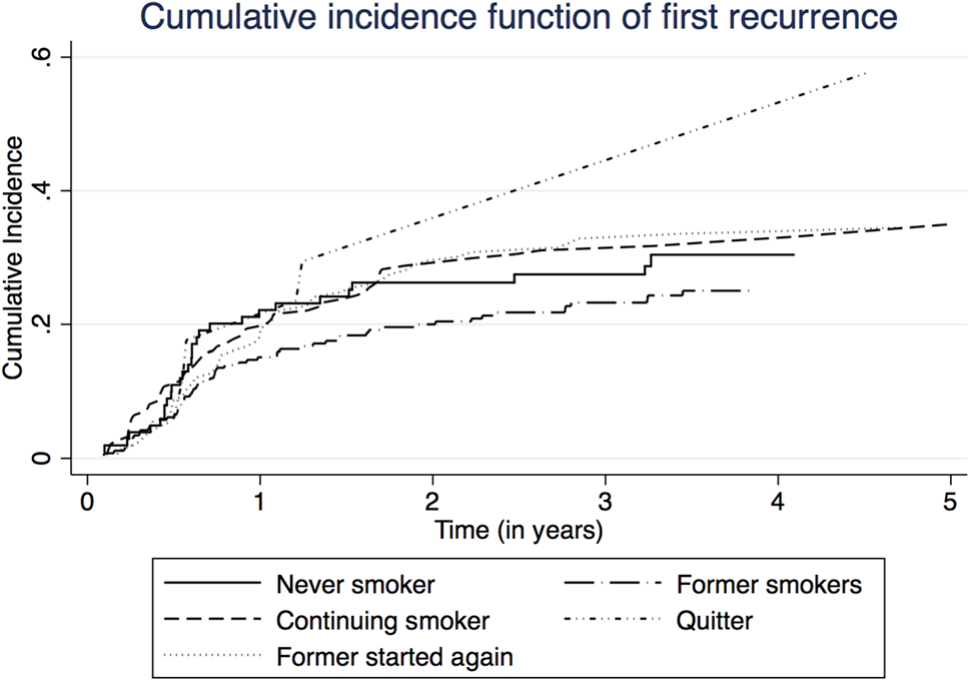
Cumulative incidence functions with correction for competing risk (death) indicating cumulative incidence of first recurrence per category of smoking status in NMIBC patients treated with TURBT

Although HR estimates for smoking cessation pre-diagnosis indicated a protective association with BC recurrence, the *p* for linear trend was not statistically significant (*p*_trend_ = 0.126) and therefore the association cannot be considered as strong (Table 2). No association between smoking status and risk of recurrence was observed in the multivariable model (Table 2). Interestingly, when compared to continuing smokers (HR 1.04, 95% CI 0.65–1.66), HRs were similar for those who quit smoking (*p* = 0.352) and former smokers who started again post-diagnosis (*p* = 0.431) (Table 2). Additionally, the cumulative incidence function shows that cumulative incidence of BC recurrence was lowest for former smokers and never smokers (Fig. 1).

**Table 2:** Cox regression analysis investigating the association between combined smoking status, smoking cessation before diagnosis and passive smoking, and time-to-first recurrence in NMIBC patients treated with TURBT

	Age and sex adjusted			Multivariable model*		
	HR	95% CI	Number of events/patients at risk	HR	95% CI	Number of events/patients at risk
Combined smoking status						
Never smoker	1.00	Ref.	29/103	1.00	Ref.	28/99
Former smoker	0.79	0.51–1.24	61/266	0.78	0.48–1.24	59/254
Continuing smoker	1.17	0.75–1.83	62/186	1.04	0.65–1.66	61/180
Former smoker who started again**	1.04	0.65–1.64	51/150	0.87	0.53–1.41	49/146
Current smoker who quit smoking***	1.25	0.52-3.00	6/17	1.47	0.63–3.41	6/17
Smoking cessation (in years)****						
< 20 years	0.81	0.46–1.43	15/48	0.82	0.46–1.46	15/47
21–40 years	0.76	0.53–1.08	57/208	0.74	0.51–1.08	54/200
> 40 years	0.67	0.44–1.02	39/155	0.71	0.46–1.09	38/148
*p* for trend	0.070			0.126		
Exposed to passive smoking during childhood?						
No	1.00	Ref.	36/142	1.00	Ref.	35/138
Yes	1.23	0.86–1.75	173/576	1.17	0.81–1.68	168/554
Exposed to passive smoking during adulthood?						
No	1.00	Ref.	74/261	1.00	Ref.	74/261
Yes	1.03	0.77–1.38	135/454	1.02	0.76–1.36	135/454

*All estimates adjusted for age, sex, stage, grade, tumor size, and number of tumors

**Former smoker who started again and current smoker who quit smoking not included in former smokers at diagnosis

***Smokers who quit after their first event are considered as current smokers

****Reference category = current smokers at diagnosis, estimates also include former smokers who started again after diagnosis

Only 25 smokers (14%) of the 174 current smokers originally recorded at diagnosis quit smoking at any point during follow-up. Three quitters were excluded for full analysis for not having information on their date last seen and another five had missing data regarding the invasiveness of their recurrent events. Of the 480 former smokers at diagnosis, 172 (36%) started smoking (any form of tobacco) again post-diagnosis in all included 846 NMIBC patients.

Exposure to environmental tobacco smoke during childhood (HR 1.17, 95% CI 0.81–1.68) or adulthood (HR 1.02, 95% CI 0.76–1.36) did not seem to have any impact on time-to-first recurrence (Table 2).

Table 3 shows HRs for time-to-first recurrence by smoking intensity, duration, and pack-years. No linear trends were observed although the highest categories showed the highest point estimates for both smoking intensity and pack-years. For smoking duration, the HRs were divergent and did not indicate any trend (*p*_trend_ = 0.729) at all.

**Table 3:** Multivariable Cox regression analysis concerning the association between smoking pack-years, intensity, and duration (recorded at diagnosis) with time-to-first recurrence in NMIBC patients treated with TURBT

	Age and sex adjusted			Multivariable model*		
	HR	95% CI	Number of events/patients at risk	HR	95% CI	Number of events/patients at risk
Never smoker	1.00	Ref.	29/103	1.00	Ref.	28/99
Pack-years						
1–9 pack-years	0.86	0.53–1.42	36/141	0.81	0.48–1.37	34/134
10–19 pack-years	0.95	0.54–1.67	22/81	0.92	0.51–1.65	22/80
20–29 pack-years	0.93	0.49–1.77	15/58	0.81	0.42–1.60	15/57
30–39 pack-years	0.70	0.35–1.43	11/55	0.60	0.30–1.22	11/53
> 40 pack-years	1.28	0.76–2.14	30/86	1.14	0.66–1.97	29/83
*p* for trend	0.365			0.688		
Smoking intensity (cigarettes/day)						
1–9 cigarettes	0.83	0.50–1.38	32/128	0.81	0.47–1.38	30/122
10–19 cigarettes	0.75	0.45–1.28	31/140	0.61	0.35–1.07	31/138
20+ cigarettes	1.24	0.79–1.96	55/167	1.16	0.72–1.85	54/160
*p* for trend	0.112			0.198		
Smoking duration (in years)						
1–9 years	1.03	0.52–2.05	12/45	0.97	0.48–1.95	12/43
10–19 years	0.94	0.54–1.62	22/83	0.85	0.48–1.50	21/78
20–29 years	0.79	0.45–1.39	21/87	0.79	0.44–1.44	20/85
30–39 years	1.08	0.61–1.89	26/88	0.93	0.52–1.66	25/85
40+ years	1.00	0.60–1.64	36/127	0.88	0.52–1.49	36/124
*p* for trend	0.917			0.729		

*All estimates adjusted for age, sex, stage, grade, tumor size, and number of tumors at diagnosis

When considering multiple events that have occurred in patients (Table 4), the HRs are similar to the time-to-first recurrence analysis (HR for continuing vs. never smokers is 1.10, 95% CI = 0.72–1.69). However, continuing smokers seemed to have experienced more recurrences than never smokers on average over 5 years on average, however not significantly (0.64 vs. 0.45, *p* = 0.308).

**Table 4:** Conditional risk set model investigating time between multiple recurrence events in NMIBC patients treated with TURBT by smoking status at diagnosis and after diagnosis

	HR*	95% CI	Number of events/patients at risk	Mean number of recurrences over 5 years (95% CI)
Smoking status				
Never smoker	1.00	Ref.	43/99	0.45 (0.28–0.63)
Former smoker	0.71	0.47–1.08	108/254	0.45 (0.33–0.57)
Continuing smoker	1.10	0.72–1.69	116/180	0.64 (0.47–0.81)
Former smoker who started again	0.89	0.56–1.43	108/146	0.82 (0.57–1.06)
Current smoker who quit smoking**	0.85	0.35–2.04	18/19	0.84 (0.10–1.58)

*All estimates adjusted for age, sex, stage, grade, tumor size, number of tumors, and re-resection of recurrent tumor

**Smokers who have quit after their first event (*n* = 2) are also included

## Discussion

### Smoking cessation post-diagnosis and BC recurrence and clinical implications

The reported HRs give reason to believe that quitting smoking does not influence the likelihood of NMIBC recurrence over 5 years when compared to continuing smokers in our sample. However, the number of quitters in our prospective sample was small which complicates drawing conclusions for this group. Another (retrospective) patient cohort study which assessed smoking cessation post-diagnosis concluded that quitting smoking significantly reduced risk of recurrence (HR 0.45, 95% CI 0.25–0.83, comparing quitters to continuing smokers); however, the proportion of quitters (~ 43% of current smokers at diagnosis) was also considerably larger [14]. In another retrospective cohort study, Fleshner et al. concluded that it remained unclear whether smoking cessation at time of diagnosis is beneficial with regard to BC recurrence [15] although Aveyard et al. estimated that the Fleshner study shows a HR of 0.71 (95% CI 0.48–1.05) when comparing quitters to continuing smokers [26], which is similar to the estimate observed in the study by Chen et al. Taken together, the limited evidence at this point seems to indicate that quitting smoking at or closely after diagnosis could reduce risk of recurrence. However, even across several smoking-related cancer sites such as lung cancer where this association is stronger, evidence to imply a strong, causal relationship between smoking behavior after diagnosis and recurrence is still limited [27]; so more prospective research is needed.

Considering the prolonged latency period for the development of BC after exposures [2], it is credible that the association between altering smoking behavior post-diagnosis and likelihood of a first recurrence or multiple recurrences over 5 years is not as strong as the association between smoking and carcinogenesis. Similarly, epidemiological evidence suggests that pre-diagnostic smoking cessation does not immediately lower the risk of BC [28], also indicating a longer latency period than 5 years. Furthermore, it is considered that a first BC recurrence is often the result of incomplete resection and/or tumor cell re-implantation, and that genuine new tumor formation only plays a more important role in later recurrences [29]. It is therefore reasonable to suggest that, because of the DNA-damaging effects of cigarette smoke [30], modifying smoking behavior may only influence later recurrences and possibly those that may occur beyond the follow-up period of 5 years reported here.

Notwithstanding the results from our study, when considering the impact of comorbidities on overall survival in BC patients [31] which include several smoking-related diseases [32] and other evidence indicating beneficial and significant results of post-diagnostic smoking cessation in retrospective studies [14, 15], it is evident that smoking cessation should be encouraged for NMIBC patients at diagnosis.

It is striking that only 14% of current smokers at diagnosis in our sample quit smoking post-diagnosis. There are examples of successful smoking cessation interventions in urology [33], and several studies found that when patients were diagnosed with BC they were more likely to quit smoking [34, 35]. Therefore, urologists should continue to improve smoking cessation counseling in newly diagnosed NMIBC patients and be updated on the available tools to improve smoking cessation figures. Moreover, more intervention clinical research investigating smoking cessation programmes in NMIBC patients is warranted.

### Smoking behavior pre-diagnosis and exposure to environmental tobacco smoke

Smoking cessation was most beneficial, with regard to reducing the risk of recurrence, the longer before diagnosis it happened compared to continuing smokers. This was the strongest association observed in our study and has been observed in other studies as well, although not consistently [12]. Other results were in line with earlier studies investigating smoking status at diagnosis and BC recurrence as well, by indicating a slightly increased risk of recurrence in NMIBC patients for current smokers compared to never smokers in a meta-analysis [10].

Another recent study not included in the aforementioned meta-analysis shows similar HRs (HR 1.49, 95% CI 0.95–2.33) for current smokers at diagnosis [8]. However, when including this study and our study (data from continuing smokers) in the meta-analysis, the pooled HR barely changes from 1.27 (95% CI 1.09–1.46) to 1.26 (95% CI 1.12–1.40) [10], indicating a significantly increased risk of recurrence for current smokers at diagnosis compared to never smokers. Possibly, the lack of association for continuing smokers in this study can be explained through multiple synchronous tumors being present at diagnosis in epithelial tumors. This theory of “field cancerization” proposes that (pre-)malignant transformation of cells has already occurred at different sites across the urothelium, explaining why (changing) smoking exposure will not have a large impact on disease prognosis [36].

Additionally, given that recent reviews indicate no considerable heterogeneity between studies that do not show an association between environmental tobacco smoke and risk of BC, it is unlikely that we would have shown any substantial association with BC recurrence either [37, 38].

Because no substantial association between smoking status pre-diagnosis and BC recurrence was observed in adjusted models it is possible that the tumor characteristics associated with BC recurrence (stage, grade, tumor size, number of tumors) included as confounders in these models overshadow the effects of smoking behavior in determining risk of BC recurrence [21] and possible also mortality since no association between quitting smoking after diagnosis and all-cause or bladder cancer-specific mortality was observed in a large retrospective cohort study [16]. Moreover, since current smokers at diagnosis in our cohort have been associated with having a higher stage, higher grade, and larger tumor size compared to never smokers [39], smoking behavior might play a more crucial role in determining risk of recurrence already before diagnosis through promoting unfavorable tumor characteristics associated with BC recurrence at diagnosis, although in a Dutch cohort of 323 UBC patients there was only a weak association between smoking intensity and increased risk of a more aggressive tumor type [40].

### Strengths and weaknesses

Despite the prospective nature of our study there were some limitations restricting the analyses. Due to the relatively short follow-up of this study, long-term effects of smoking cessation post-diagnosis could not be assessed and the number of deaths due to BC in the NMIBC patients within our cohort was too low for Cox regression analysis. Also, it was not possible to obtain detailed information on adjuvant therapy for all patients, so differences in adjuvant therapy could not be considered in the statistical analysis. Additionally, we did not correct for biomarkers of BC recurrence such as mutations in the *FGFR3* or *TP53* genes [41], although they might work together with smoking intensity in predicting BC outcome [42].

Furthermore, one of the caveats of using only self-reported questionnaire data to assess smoking exposure was likely demonstrated in our sample of NMIBC patients. The large proportion (about 1 in 3) of former smokers pre-diagnosis who reported to have started smoking again post-diagnosis is implausible and is probably observed due to misclassification of either the questionnaire at baseline or during follow-up. A high misclassification rate (47%) when comparing self-reported data on smoking behavior to cotinine values in blood was also shown in another sample of bladder cancer patients undergoing surveillance [43]. Preferably, future studies should consider more reliable ways of verifying smoking exposure through biochemical analysis.

Unfortunately, at the start of the study we did not anticipate this small proportion of quitters after diagnosis which is why the analysis concerning quitters is underpowered.

## Conclusion

Although quitting smoking after diagnosis might reduce probability of recurrence based on retrospective evidence, the number of NMIBC patients quitting smoking in our prospective study was low. This indicates an important role for urologists and other health care professionals in promoting smoking cessation in NMIBC. Based on the current evidence, smoking cessation pre-diagnosis seems to have the largest impact on reducing risk of recurrence after NMIBC diagnosis.
